# The influence of social support on COPD outcomes mediated by depression

**DOI:** 10.1371/journal.pone.0245478

**Published:** 2021-03-17

**Authors:** Leonard Turnier, Michelle Eakin, Han Woo, Mark Dransfield, Trisha Parekh, Jerry A. Krishnan, Richard Kanner, Christopher B. Cooper, Prescott G. Woodruff, Robert Wise, MeiLan K. Han, Karina Romero, Laura M. Paulin, Stephen Peters, Brad Drummond, Eugene R. Bleecker, Russell Bowler, Alejandro P. Comellas, David Couper, Robert Paine, Fernando Martinez, Graham Barr, Nirupama Putcha, Nadia N. Hansel

**Affiliations:** 1 Johns Hopkins Department of Medicine, Johns Hopkins University School of Medicine, Baltimore, Maryland, United States of America; 2 University of Alabama Birmingham, Birmingham, Alabama, United States of America; 3 University of Illinois, Chicago, Illinois, United States of America; 4 University of Utah School of Medicine, Salt Lake City, Utah, United States of America; 5 University of California at Los Angeles, Los Angeles, California, United States of America; 6 University of California at San Francisco, San Francisco, California, United States of America; 7 University of Michigan, Ann Harbor, Michigan, United States of America; 8 Wake Forest Baptist Health, Winston-Salem, North Carolina, United States of America; 9 University of North Carolina School of Medicine, Chapel Hill, North Carolina, United States of America; 10 National Jewish Health, Denver, Colorado, United States of America; 11 University of Iowa Carver College of Medicine, Iowa City, Iowa, United States of America; 12 University of North Carolina, Gillings School of Global Public Health, Chapel Hill, North Carolina, United States of America; 13 Weill Medical College, New York, New York, United States of America; 14 Columbia University Medicine Center, New York, New York, United States of America; University of Sao Paulo Medical School, BRAZIL

## Abstract

**Background:**

The purpose of this study was to explore the association between perceived social support and COPD outcomes and to determine whether the associations are mediated by depressive symptoms.

**Methods:**

Subjects with COPD who were enrolled as part of SPIROMICS were included in this analysis. Questionnaires relating to quality of life, symptom burden, and functional status were administered at annual clinic visits for over a 3 year period. In both cross-sectional and longitudinal analyses, we examined the association of social support as measured by the FACIT-F with COPD outcomes. Cross sectional analyses used multivariable linear or logistic regression, adjusting for covariates. For longitudinal analyses, generalized linear mixed models with random intercepts were used. Models were adjusted with and without depressive symptoms and mediation analyses performed.

**Results:**

Of the 1831 subjects with COPD, 1779 completed the FACIT- F questionnaire. In adjusted cross-sectional analysis without depressive symptoms, higher perceived social support was associated with better quality of life, well-being, 6 minute walk distance, and less dyspnea. When also adjusting for depressive symptoms, all associations between social support and COPD outcomes were attenuated and no longer statistically significant. Mediation analysis suggested that depressive symptoms explained the majority (> = 85%) of the association between social support and measured COPD outcomes. Results of the longitudinal analysis were consistent with the cross-sectional analyses. There was no association between social support and odds of exacerbations.

**Conclusion:**

Higher social support was associated with better COPD outcomes across several measures of morbidity including quality of life, respiratory symptoms, and functional status. In addition, these associations were largely attenuated when accounting for depressive symptoms suggesting that the beneficial association of social support with COPD outcomes may be largely mediated by the association between social support and depression.

**Trial registration:**

SPIROMICS was approved by Institutional Review Boards at each center and all participants provided written informed consent (clinicaltrials.gov: NCT01969344).

## Introduction

Chronic Obstructive Pulmonary Disease (COPD) is a common chronic illness that affects approximately 16 million Americans [1]. Most patients who suffer from this progressive disease report shortness of breath, cough and phlegm, and exercise limitation. These debilitating symptoms can heavily influence daily activities [2], and patients become susceptible to restrictions on employment, recreation, and social activity; and, they may endure hardships in social relationships and within social networks [3]. Having positive social support has been associated with positive outcomes in other chronic diseases [4, 5]; but studies in COPD have been few with existing studies evaluating only few measures of COPD morbidity, many limited by small sample size, being cross-sectional in nature, and thus limiting definitive conclusions. Perceived social support has been inversely associated with depression in patients with COPD [6, 7], and depression is a common comorbidity among COPD patients [8], with a broad impact on health outcomes, with higher depressive symptoms linked to decreased functional status, impaired quality of life, and higher mortality in patients with COPD [8].

The objective of this analysis is to evaluate the association between perceived social support and COPD outcomes, including quality of life, respiratory symptoms, functional status and exacerbation risk, and the potential mediating role of depressive symptoms, using the large multi-center study of Subpopulations and Intermediate Outcome Measures In COPD Study (SPIROMICS), a multi-site observational study that followed people with COPD for up to three years.

## Methods

### Study design

SPIROMICS participants were between the ages of 40–80 years, recruited from twelve clinic sites. Some non-smoking controls were recruited, but the majority had a smoking history of 20 or more pack-years with and without COPD. The goal of this investigation is to understand the role of social support among adults with COPD, thus only those with history of smoking and COPD were included in this analysis and were defined based on the presence of airways obstruction (i.e., Forced Expiratory Volume_1_ FEV_1_ /Forced Vital Capacity (FVC) less than 0.7; FEV_1_ < 80%). Participants performed spirometry according to American Thoracic Society criteria [9]. Questionnaires were administered that assessed demographics, smoking history, respiratory symptoms, quality of life, depressive symptoms, and social support. Participants were followed annually with ascertainment of depressive symptoms, social support and respiratory outcomes (symptoms, quality of life) yearly for up to 3 years, allowing for cross-sectional and longitudinal analyses.

SPIROMICS was approved by Institutional Review Boards at all respective clinical sites (Johns Hopkins University, Columbia University, University of Utah, University of California San Diego, National Jewish Hospital, University of Alabama Birmingham, University of North Carolina, Wake Forest University, University of Michigan, Temple University, University of Illinois, University of Iowa and University of California Los Angeles). All participants provided written informed consent (clinicaltrials.gov: NCT01969344).

### Social support

Perceived social support was measured using the Social/Family Well-Being domain of the self-reported Functional Assessment of Chronic Illness Therapy- Fatigue questionnaire [10]. The domain consisted of 8 questions that ascertained a participant’s current relationship to family, friends, and significant other, with a sum score between 0–28 with a higher score indicative of higher social support. Negatively phrased questions within the domain were reverse scored for the total sum score.

### Depressive symptoms

The Hospital Anxiety and Depression questionnaire is a self-reported scale with seven questions that specifically pertain to depressive symptoms with scores ranging between 0–21 with any score 8 or above representing mild to severe symptoms of depression [11, 12].

### Outcomes

Outcomes of interest included respiratory-specific questionnaires encompassing quality of life (St. George’s Respiratory Questionnaire, SGRQ) a disease-specific instrument measuring quality of life using 50 items in three subscales (symptoms, activity, and impact) [13], impact of COPD (COPD Assessment Test, CAT), an 8-item, sensitive, valid, reliable, and standardized measure of COPD global health status impairment used in COPD severity classification guidelines [14] and dyspnea (Modified Medical Research Council Dyspnea Scale, mMRC) [15] a 1-item scale validated for categorizing patients in terms of disability attributable to dyspnea. Exercise capacity was measured using a six minute walk distance in meters (6MWD) [16].

At baseline, exacerbations in the previous year were based upon participant report of medication changes or dose adjustments, unscheduled doctor visits, emergency room visits and hospitalizations for COPD exacerbations and frequency of these instances over the past year. Severe exacerbations in the previous year were defined as events requiring emergency room visit or hospitalization. These counts were dichotomized to represent any or none for our analytic exacerbation outcome. For the longitudinal analysis, an equivalent yearly count of exacerbations was used—dichotomized to indicate any event or none in the past year which were self-reported by each patient during quarterly phone calls.

### Statistical analysis

The descriptive analyses were run to examine means and proportions. Participant characteristic and outcomes for high and low social support were compared based on dividing the sample into above and below-median FACIT social and family well-being domain, using t-tests and chi-squared tests for continuous and categorical variables.

The independent cross-sectional association between social support and outcomes was analyzed using multivariable linear or logistic regression, adjusted by age, gender, race (white vs. non-white), body mass index (BMI), marital status (married vs. not married), level of education (some college or above vs. high school graduate or below), household income (less than $50,000, more than or equal to $50,000, and declined to answer), current smoking status, pack-years, and FEV_1_ percent predicted. These variables were chosen based upon past findings concerning social support and COPD outcomes. Models were also adjusted with and without depressive symptoms as a part of the mediation analysis—described later in more detail.

To assess the longitudinal association (controlling for time trend and random participant effect) between social support and outcomes, we used generalized linear mixed models with random intercepts for participants. All covariates in the cross-sectional model were included as time-fixed baseline covariates except for depressive symptoms, which varied across visits and time, which was modeled continuously based on visit year. In follow up data, there was missingness due mainly to the ending of study funding before participants could complete their follow-up visits. We conducted listwise and multiple imputation analysis under missing-completely-at-random (MCAR) and missing-at-random (MAR) assumptions respectively. The detailed description of missing analysis, along with multiple imputation results, is included in the online supplement.

### Mediation analysis

**Fig 1:**
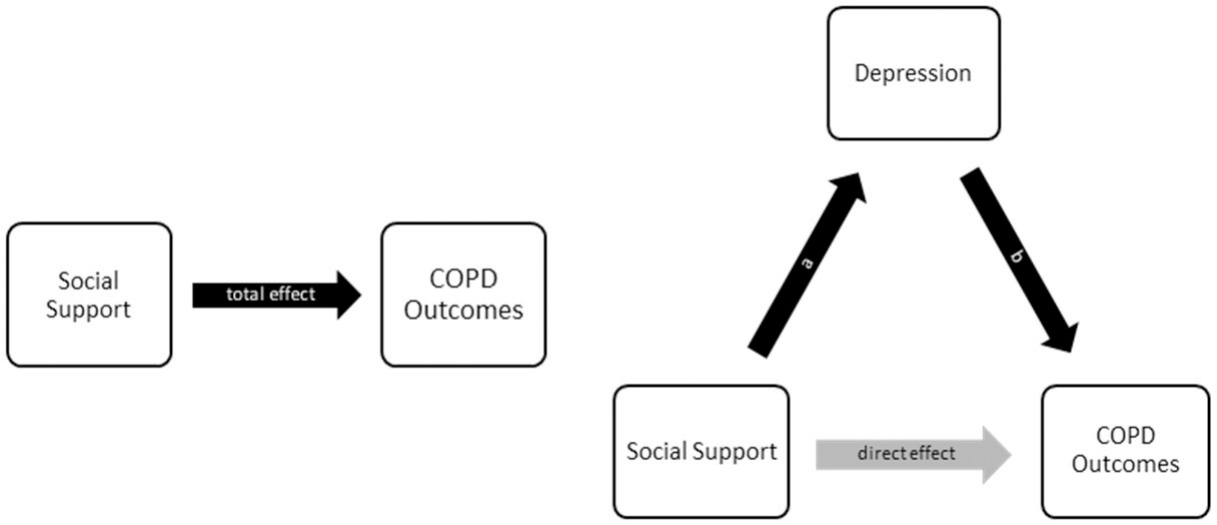
Mediation proportion as the percentage of total effect “Explained” (or Reduced) by the mediator, or equivalently, 1 –direct effect/total effect = mediation proportion.

We tested the interaction between social support and depressive symptom and found no strong evidence to suggest that depressive symptom moderates the association between social support and outcomes. We therefore proceeded with mediation analysis which followed the standard approach based on the method of Baron and Kenny [17]. The presence of depressive symptoms was considered a mediator if the following four conditions were met: 1) social support was associated with outcome, 2) social support was associated with depressive symptoms, 3) depressive symptoms were associated with outcomes when controlling for social support, and 4) the association between social support and outcome in Step 1 was significantly attenuated when depressive symptoms were included as a covariate. For outcomes that did not meet the first three conditions, mediation analysis was not conducted. The relative size of mediation in relation to total effect (i.e., the percentage of social effect explained by depressive symptoms) was conceptualized as “mediation proportion” and operationalized as the following: *mediation proportion = 1 –direct effect / total effect* (Fig 1). Mediation proportion was allowed to exceed 100% where the signs of social support-outcome associations were reversed following depression adjustment, as well as below 0% where there was proportional gain instead of reduction following depression adjustment.

For the longitudinal analysis, a linear mixed model with random intercept was used within the above procedure. All steps were run in both unadjusted and adjusted model settings. The conventional terminology of mediation analysis—for example, “total effect”, “direct effect”, and “indirect/mediated effect”—are used with the understanding that “effect” refers to “effect estimates”.

All analyses were conducted with Stata 15.1 [18]. All confidence interval of sample statistics in regression models, including mediation proportion in mediation analysis, were generated based on bootstrapping approach [19]. The threshold for statistical significance was p<0.05, based on 95% confidence interval.

## Results

### Participant characteristics

Of the 1831 subjects with COPD, 52 subjects did not complete the FACIT-F questionnaire and thus 1779 (97%) were included in the final analysis. The mean level of social support was 20.2 (SD 6.1) with the median of 21 (IQR 8). As shown in Table 1, those with higher social support (above median) were more likely to be older, married, report yearly earnings greater than $50,000, less likely to be currently smoking, and less likely to be high school graduate or above. Those with higher social support were also less likely to have depressive symptoms in all categories (mild, moderate, and severe). All differences were statistically significant (P<0.05).

**Table 1:** Participant characteristic.

		By Social Support		
	All (N = 1779)	Low (N = 953)	High (N = 826)	
	Mean ± SD or %	Mean ± SD or %	Mean ± SD or %	P-value
**Demographics**				
Age	65.1 ± 8.1	64.1 ± 8.2	66.2 ± 7.8	**<0.001**
Female %	43	40	45	0.055
White %	81	82	80	0.332
Some college or above %	61	64	58	**0.019**
Married %	49	43	58	**<0.001**
Income				**0.002**
< 50k %	51	55	46	
> = 50k %	31	29	34	
Decline to answer %	18	17	20	
Currently smoking %	34	38	29	**<0.001**
BMI	27.3 ± 5.3	27.2 ± 5.3	27.4 ± 5.4	0.452
Packyears	52.7 ± 27.5	52.8 29.2	52.2 ± 24.5	0.860
**Pulmonary Function**				
FEV1% Pred.	53.4 ± 22.4	53.7 ± 22.7	52.6 ± 22.0	0.286
**COPD-related outcomes**				
SGRQ Overall	38.0 ± 19.8	40.3 ±20.5	35.7 ± 18.5	**<0.001**
CAT	15.4 ± 8.0	16.2 ± 8.1	14.6 ± 7.8	**<0.001**
6MWD	392 ± 127	390 ± 126	395 ± 128	0.685
MMRC (0–4 cont.)	1.26 ± 1.04	1.34 ± 1.06	1.17 ± 1.00	**<0.001**
Base. Exac. % (Yes/No)	32	31	33	0.351
Base. Sev. Exac. % (Yes/No)	16	15	17	0.367
**Social Support**				
FACIT (cont.)	20.1 ± 6.1	15.7 ± 4.8	25.3 ± 2.2	**<0.001**
**Depression**				
HADS (cont.)	4.7 ± 3.5	5.6 ± 3.8	3.5 ± 2.8	**<0.001**
HADS (categorical)				
None %	80	71	89	**<0.001**
Mild %	13	18	8	**<0.001**
Moderate %	6	9	2	**<0.001**
Severe %	1	2	0.1	**<0.001**

"Low" and "High" social support is based on quantile FACIT score, with "Low" indicating the below-median and "High" the above-median.

St. George’s Respiratory Questionnaire (SGRQ); COPD Assessment Test (CAT); SIx Miniute Walking Distance (6MWD); Forced Expiratory Volume (FEV1); Modified Medical Research Council (mMRC); Hospital Anxiety and Depression Scale (HADS).

mMRC was measured on a continuous scale; HADS (categorical) used a single chi-squared test.

With respect to outcomes, those with higher social support had better quality of life and well-being with lower SGRQ and CAT scores and less dyspnea (MRC); all P<0.05. They did not show significant differences in lung function or exacerbation risk.

### Bivariate and multivariable regression results

In unadjusted models, every one standard deviation (SD) increase in social support (~ 6 point increase FACIT scale) was associated with significantly lower SGRQ score (β, -2.9; 95% CI: -3.9, -1.8), CAT score (β, -1.0; 95% CI: -1.4, -0.6), and dyspnea score (β, -0.1; 95% CI: -0.15, -0.05). Exacerbation risk and 6MWD were not associated with social support. In adjusted models including all covariates except for depressive symptoms (Table 2, **M1**), the association between higher social support and health outcomes remained similar. In addition, social support’s association with 6MWD reached statistical significance. Specifically, for every one SD increase in social support, SGRQ, CAT, and dyspnea scores were lower by 2.3, 0.8, and 0.1 points respectively, while 6MWD was higher by 8.2 meters (all P<0.05). There remained no association between social support and exacerbation risk.

**Table 2:** Baseline regression of COPD outcomes mediated by depression.

	M1	M2	Mediation %
	β 95% Cl	β 95% Cl	β 95% Cl
SGRQ Overall (Points)	-2.32* -3.16 -1.44	0.41 -0.46 1.19	117%* 85% 179%
CAT (Points)	-0.77* -1.16 -0.41	0.25 -0.09 0.58	132%* 91% 227%
6MWD (m)	8.17* 1.74 14.35	1.22 -5.53 7.38	85%* 40% 328%
mMRC (0–4)	-0.09* -0.14 -0.05	0.00 -0.04 0.04	100%* 66% 187%
	OR 95%Cl	OR 95%Cl	OR 95% Cl
Exacerbation (Y/N)	1.03 0.92 1.17	1.10 0.97 1.27	
Severe Exacerbation (Y/N)	1.05 0.91 1.22	1.13 0.97 1.33	

• M1: Adjusted model *without* depression; M2: Adjusted model *with* depression; Mediation % = (βM1 − βM2)/ βM1.

• All models are adjusted by age, gender, race, education, income, marital status, BMI, smoking status, packyears, and FEV1% predicted; confidence intervals are generated based on bootstrapping approach.

• For modeling approach, least squares regressions was used for continuous outcomes (SGRQ, CAT, 6MWD, and MRC), and logistic regression was used for binary outcomes (exacerbation and severe exacerbation at baseline).

• * Indicates bootstrap p-value of <0.05 (or, statistical significance at 95% confidence interval).

**Fig 2:**
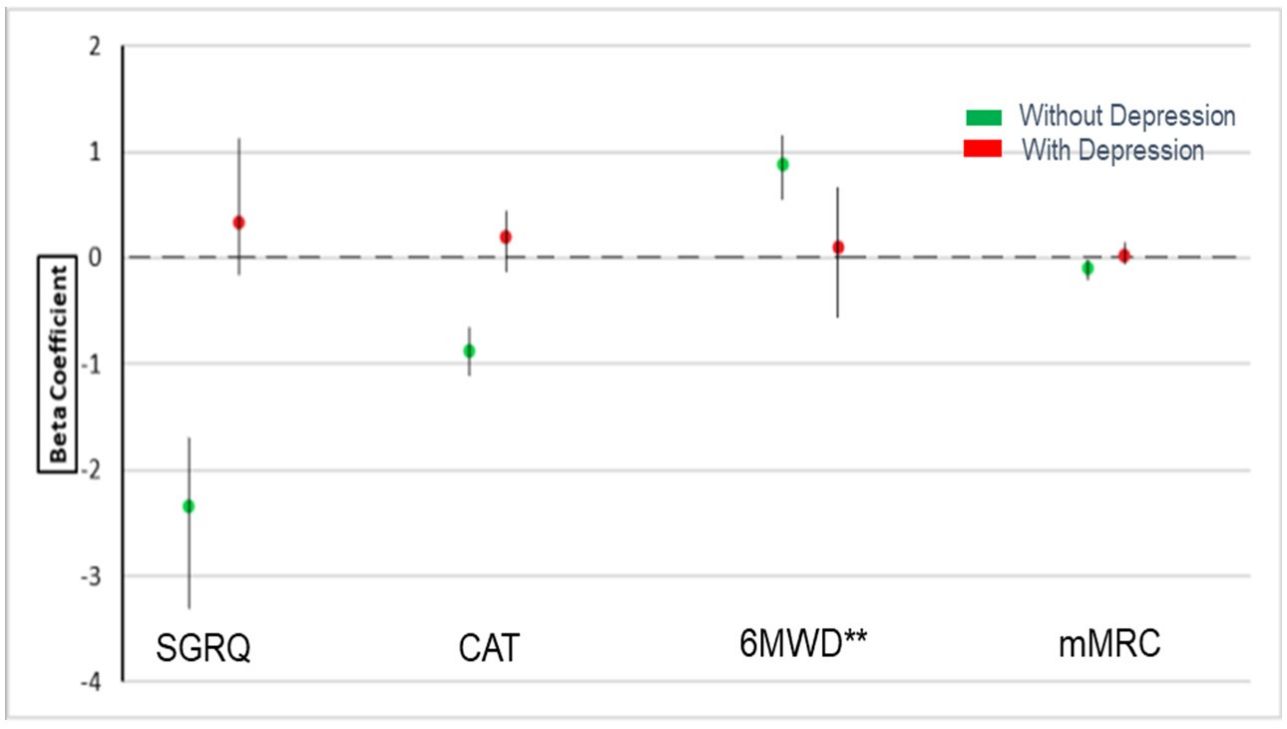
Association of social support on COPD outcomes with/without depression.

When depressive symptoms were included as a covariate in the models, the associations between social support and COPD health outcomes were substantially attenuated and for some the effect estimate changed direction. Social support was no longer statistically significantly associated with any measured health outcomes (Table 2, **M2**; Fig 2).

### Mediation results

There was a statistically significant association between social support and depressive symptoms, with one SD increase in social support associated with 1.1 point reduction in HADS-D depression scale (β, -1.1; 95% CI: -1.3, -1.0). Individuals with severe COPD were more likely to report depressive symptoms in comparison to those with mild COPD (27 vs. 17%, P<0.001). Mediation analysis for exacerbations was not performed since social support was not statistically significantly associated with exacerbation risk and failed to meet the criteria for mediation. For all other outcomes, depressive symptoms either fully or nearly fully attenuated the association of social support with outcomes, with statistically significant mediated or indirect effect (all P<0.05) and the amount of explanation—or mediation proportion—ranging from 85% to 130% (Table 2). For example, one SD increase in social support was associated with an 8.2 meter increase in the predicted level of 6MWD (β, 8.2, 95% CI: 1.7, 14.4), but, when adjusting by depressive symptoms, only a 1.2 meter increase and it was no longer statistically significant (β, 1.2; 95% CI: -5.5, 7.8). The reduction in beta from 8.2 to 1.2 represented 85% mediation, which was statistically significant (95% CI: 40%, 328%) (Table 2).

The longitudinal analyses showed similar patterns as the baseline analysis except that 6MWD was not significantly associated with social support in adjusted models without depression. Similar to cross-sectional analyses, higher social support showed significant association with better outcomes in SGRQ, CAT, and MMRC (all P<0.05, Table 3, **M1**), but these associations were substantially attenuated when adjusting by depressive symptoms. The results for mediation analysis remained similar. Social support was significantly related to depressive symptoms, with every one SD increase in social support associated with about 1 point reduction in HADS depression scale (β, -0.95; 95% CI: -1, -0.7); likewise, depressive symptoms were significantly associated with measured health outcomes (data not shown). For outcomes satisfying mediation criteria (SGRQ, CAT, and MMRC), social support’s indirect effects were statistically significant (P<0.05), and were almost fully explained by depressive symptoms (ranging from 88% to 96% mediation), with none of their direct effects remaining statistically significant after adjusting for depressive symptoms (Table 3). For example, every one SD increase in social support was associated with a 0.8 lower CAT score (β, -0.8; 95% CI: -1.0, -0.5), but depressive symptoms mediated this association by 88% (95% CI: 57%, 111%), and social support was no longer statistically significantly associated with CAT after adjusting by depressive symptoms (β, -0.1; 95% CI: -0.4, 0.1).

**Table 3:** Longitudinal analysis with all available cases.

	M1	M2	Mediation %
	β 95% Cl	β 95% Cl	β 95% Cl
SGRQ Overall (Points)	-1.43* -1.81 -0.80	-0.10 -0.66 0.32	93%* 65% 140%
CAT (Points)	-0.79* -0.98 -0.51	-0.10 -0.41 0.06	88%* 57% 111%
6MWD (m)	3.66 -1.60 5.91	-0.97 -5.84 2.25	
mMRC (0–4)	-0.09* -0.11 -0.05	0.00 -0.05 0.02	96%* 57% 133%
	OR 95%Cl	OR 95%Cl	OR 95% Cl
Exacerbation (Y/N)	1.02 0.90 1.12	1.12 0.98 1.37	
Severe Exacerbation (Y/N)	1.01 0.82 1.29	1.11 0.89 1.32	

• M1: Adjusted model *without* depression; M2: Adjusted model *with* depression; Mediation % = (βM1 − βM2)/ βM1.

• All models are adjusted by age, gender, race, education, income, marital status, BMI, smoking status, packyears, and FEV1% predicted; confidence intervals are generated based on bootstrapping approach.

• For modeling approach, generalized linear mixed model (GLMM) with identity and logit link were used for continuous and binary outcomes respectively, with random participant effect.

• * Indicates bootstrap p-value of <0.05 (or, statistical significance at 95% confidence interval).

## Discussion

To our knowledge, this is one of the largest population used to evaluate the association of social support and COPD morbidity and the first to consider whether effects of social support on COPD outcomes are mediated by depressive symptoms. Our results show that higher social support is associated with better COPD outcomes across several measures of morbidity, including better quality of life, fewer respiratory symptoms and higher functional status. Further, the link between social support and better COPD outcomes was largely mediated by depressive symptoms, and these results were consistent across cross-sectional and longitudinal analyses.

The mean level of social support in our cohort was 20.1 (SD 6.1), which overall reflects similar degree of social support compared to the general population average of 19.9 (SD 4.8) from previous literature [10]. Our study adds substantially to the literature, as studies to date investigating the effects of social support on COPD outcomes have focused primarily on depression or quality of life as the outcomes, have been cross-sectional with many having small sample size, limiting definitive conclusions. A few small studies showed no significant association between social support and QOL [12, 16, 20]; however, one larger study conducted in Sweden suggested that individuals who reported persons in the vicinity who can provide personal support in case of personal problems or life crises reported better QOL [21]. Our results support the hypothesis that perceived social support is positively associated with respiratory-specific quality of life as measured by SGRQ, and expands significantly upon current knowledge, by investigating the association of social support on multiple measures of COPD morbidity, in a large well-characterized cohort of subjects with COPD. In particular, social support was also significantly associated with CAT and mMRC, which reflect respiratory symptoms and are recommended instruments in Global Initiative for Chronic Obstructive Lung Disease (GOLD) Guidelines to guide therapeutic management [22].

To our knowledge, there have been no studies considering whether the influence of social support on COPD outcomes is mediated by depressive symptoms, despite consistent evidence that shows increased social support is linked with fewer symptoms of depression in COPD [6, 7, 21] and other chronic diseases. Depressive symptoms, in turn, significantly influence patient-reported outcomes, physical health status, and exercise capacity [23, 24]. Within our population, 21.4% of patients had depressive symptoms ranging from mild to severe, consistent with previous studies of COPD [25]. Our results show a strong association between higher social support and lower depressive symptoms, and our mediation analyses show that depressive symptoms largely mediate the association of social support on several measured health outcomes. Specifically, in cross-sectional and longitudinal analyses, the effects of social support on SGRQ, CAT and MMRC was almost fully mediated by depressive symptoms.

Our study has several limitations which should be noted. Our study was based on a single measure of emotional and informational social support, but social support can be identified in several ways. Types of social support can differ from emotional, instrumental (services or materials to help with day to day activities), appraisal (self- evaluation), and informational support (giving information or advice) [26]; thus we may have missed associations attributed to appraisal or instrumental support. An important limitation is that social support was assessed by self-report questionnaire and thus is dependent on perceived social support which may be affected by whether one suffers from depressive symptoms, thus the strong association between social support and depressive symptoms may be bi-directional. Further work assessing objective measures of social support may be necessary to confirm our findings. It is notable that these mediation analyses are correlational in a nature and it is possible that some other third variable, independent from the proposed mediator of depressive symptoms, could be responsible for the proposed effect. For example, there are several additional pathways by which social support may be linked to better COPD outcomes. These include better adherence to medication regimen, improved self-management, and enhanced self-efficacy and self-care behaviors [27–29], and social support has been associated with self-care behaviors and self-efficacy in patients with COPD [11, 12]. We were unable to assess additional potential behavioral mechanisms by which social support may affect outcomes. Despite the consistent link between social support and COPD outcomes, which was almost fully mediated by depressive symptoms, there were no associations between social support and exacerbation risk. Though this is an unexpected finding, this may be explained by individuals with more social support being more likely to seek medical care and receive treatment for worsened symptoms. In terms of generalizability concerning other populations, a limitation would be that most of the patients within the study are white and findings of the study associated with other cultures and countries is unknown. An additional concern is the substantial number of missing visits in our longitudinal analysis, however the benefit of showing consistent results in both cross-sectional and longitudinal analysis—including multiple imputation analyses—lends support to a robust link between social support and COPD outcomes, with effects mediated by depressive symptoms.

## Conclusion

Our results show that increased social support was significantly associated with lower odds of depressive symptoms, which in turn was linked with better COPD outcomes. Importantly, the identified health benefits of higher social support were predominantly mediated by depressive symptoms. There is a high prevalence of depressive symptoms among patients with COPD and depressive symptoms are associated with multiple COPD morbidity measures. Given the large variability in degrees of existing social support, studies are needed to determine whether interventions to increase social support in patients with COPD may have additional positive impact on depressive symptoms in addition to standard therapies, including medication and cognitive behavioral therapy, and thus may subsequently lead to improved COPD outcomes.

## Supporting information

S1 Appendix The influence of social support on COPD outcomes mediated by depression. (DOCX)

S1 Table Baseline multiple imputation + bootstrap mediation analysis, based on bootstrap nested within multiple imputation. (XLSX)

S2 Table Longitudinal multiple imputation + bootstrap mediation analysis, based on bootstrap nested within multiple imputation. (XLSX)

## Abbreviations

6MWD: Six Minute Walk Distance
CAT: COPD Assessment Test
COPD: Chronic Obstructive Pulmonary Disease
FACIT-F: Functional Assessment of Chronic Illness Therapy- Fatigue
FEV1: Forced Expiratory Volume
FVC: Forced Vital Capacity
GOLD: Global Initiative for Chronic Obstructive Lung Disease
HADS: Hospital Anxiety and Depression Questionnaire
MAR: Missing At Random
MCAR: Multiple Imputation Analysis under missing-completely-at-random
mMRC: Modified Medical Research Council Dyspnea Scale
SD: Standard Deviation
SGRQ: St. George’s Respiratory Questionnaire
SPIROMICS: Subpopulations and Intermediate Outcome Measures In COPD
STATA: Software for Statistics and Data Science
