# Supplementary material for: The influence of social support on COPD outcomes mediated by depression
Source: PLoS One. 2021 Mar 17;16(3):e0245478. doi: 10.1371/journal.pone.0245478 (PMC7968645; doi:10.1371/journal.pone.0245478)
Supplement: S1 Appendix — (DOCX) [file pone.0245478.s001.docx]

**S1 Appendix: The Influence of Social Support on COPD Outcomes Mediated by Depression**

***Missing Analysis***

The missing pattern in our longitudinal sample followed the monotone pattern, with overall ~ 33% missingness per wave (specifically, 20% missing from baseline to first follow-up, 36% missing from first follow-up to the second, and 42% missing from second follow-up to the third), which is within the range for which either listwise or multiple imputation analysis is shown to handle if either MCAR or MAR assumption hold respectively[R1]. Given that the primary reason for the missingness in our data is study funding and given that funding is exogenous to participants’ characteristics, both MCAR and MAR assumptions are plausible. Accordingly, we present the listwise analysis under MCAR assumption (in the main text), as well as multiple imputation analysis with MAR assumption here in the online supplement.

As all our regression analysis in this paper utilized the bootstrap approach, for the missing analysis we combined the multiple imputation with bootstrap approach to generate the confidence interval for the point of estimates of our interest. We followed the “MI Boot” or “bootstrapping nested within multiple imputation” procedure recommended in the literature[R2, R3]. First, missing observations were imputed using all of our analytic variables as predictors--based on multivariate imputation by chained equation (MICE); second, for each imputed dataset, bootstrap analysis were run. In the imputation stage, clinical outcomes and exacerbation outcomes were separated. Given the fraction of missing in our data, we set the number of imputation at 30[R2, R3]. All bootstrap analyses were conducted with 1000 replication with replacement.[R2, R3] Type 1 error rate was set at 5% critical level, based on 95% bootstrap percentile confidence interval.

The table below shows side by side the information on missing fraction of our sample. And the last two tables show the results of the complete case analysis of baseline and longitudinal data based on the procedure described above.

| **Baseline Stratum 3, 4 (N=1831)** | | | |
| --- | --- | --- | --- |
|  | Freq. | % |  |
| ID | 0 | 0 |  |
| VISIT | 0 | 0 |  |
| AGE | 0 | 0 |  |
| FEMALE | 0 | 0 |  |
| RACE | 0 | 0 |  |
| EDUCATION | 4 | 0.2 |  |
| INCOME* | 0 | 0 |  |
| MARITAL STATUS | 3 | 0.2 |  |
| BMI | 0 | 0 |  |
| SMOKING STATUS | 30 | 1.6 |  |
| PACKYEARS | 1 | 0.1 |  |
| FEV % Pred. | 3 | 0.2 |  |
| SOCIAL SUPPORT | 52 | 2.8 |  |
| DEPRESSION | 24 | 1.3 |  |
| SGRQ | 115 | 6.3 |  |
| CAT | 82 | 4.5 |  |
| 6MWD | 102 | 5.6 |  |
| MMRC | 15 | 0.8 |  |
| Excerbation | 21 | 1.1 |  |
| Severe Exacerbation | 25 | 1.4 |  |
|  |  |  |  |
| **18 participants out of 1831 were missing income; they were included along with 327 participants who answered "Don't know".* | | | |

| **Longitudinal Stratum 3, 4 (N=7324)** | | | |
| --- | --- | --- | --- |
|  | Freq. | % |  |
| ID | 0 | 0 |  |
| VISIT | 0 | 0 |  |
| AGE | 0 | 0 |  |
| FEMALE | 0 | 0 |  |
| RACE | 0 | 0 |  |
| EDUCATION | 16 | 0.2 |  |
| INCOME* | 0 | 0 |  |
| MARITAL STATUS | 12 | 0.2 |  |
| BMI | 0 | 0 |  |
| SMOKING STATUS | 120 | 1.6 |  |
| PACKYEARS | 4 | 0.1 |  |
| FEV % Pred. | 12 | 0.2 |  |
| SOCIAL SUPPORT | 2738 | 37.4 |  |
| DEPRESSION | 2676 | 36.5 |  |
| SGRQ | 2945 | 40.2 |  |
| CAT | 2804 | 38.3 |  |
| 6MWD | 2970 | 40.6 |  |
| MMRC | 2657 | 36.3 |  |
| Excerbation | 2621 | 35.8 |  |
| Severe Exacerbation | 2625 | 35.8 |  |
|  |  |  |  |

**An additional table file shows this in more detail [see Additional S1 Table**].

“Baseline Multiple Imputation + Bootstrap Mediation Analysis, based on Bootstrap nested within Multiple Imputation”.

**An additional table file shows this in more detail [see Additional S2 Table]**

“Longitudinal Multiple Imputation + Bootstrap Mediation Analysis, based on Bootstrap nested within Multiple Imputation”

**REFERENCES:**

[R1] D. Newman, "Longitudinal Modeling with Randomly and Systematically Missing Data: A Simulation of Ad Hoc, Maximum Likelihood, and Multiple Imputation Techniques," *Organizational Research Methods - ORGAN RES METHODS,* vol. 6, pp. 328-362, 07/01 2003.

[R2] M. Schomaker and C. Heumann, "Bootstrap inference when using multiple imputation," (in eng), *Stat Med,* vol. 37, no. 14, pp. 2252-2266, Jun 30 2018.

[R3] J. Brand, S. van Buuren, S. le Cessie, and W. van den Hout, "Combining multiple imputation and bootstrap in the analysis of cost-effectiveness trial data," (in eng), *Stat Med,* vol. 38, no. 2, pp. 210-220, Jan 30 201
